# α-Tocopherol/Gallic Acid Cooperation in the Protection of Galactolipids Against Ozone-Induced Oxidation

**DOI:** 10.1007/s00232-015-9851-4

**Published:** 2015-10-24

**Authors:** Elżbieta Rudolphi-Skórska, Maria Filek, Maria Zembala

**Affiliations:** Department of Biochemistry, Biophysics and Biotechnology, Institute of Biology, Pedagogical University, Podchorążych 2, 30-084 Kraków, Poland

**Keywords:** Oxidation, Antioxidants, Gallic acid, α-Tocopherol, Galactolipids

## Abstract

The protective ability of α-tocopherol (TOH) and gallic acid (GA) acting simultaneously at the moment of oxidizer application was evaluated by determination of galactolipid layers’ oxidation degree. Addition of GA resulted in a significant decrease of ozone-derived radicals shifting the threshold of lipid sensitivity by an amount approximately corresponding to the GA intake in bulk reaction with ozone. TOH presence in lipid layers results in a change of the role of GA which additionally may be involved in the reduction of tocopheroxyl radical formed during oxidation. This leads to a decrease in effectiveness of GA in diminishing the amount of ozone radicals. Such an effect was not observed for mixed layers containing galactolipid and pre-oxidized tocopherol where the ozone threshold level was associated with a stoichiometry of GA + O_3_ reaction. It was concluded that probably subsequent transformations of tocopheroxyl radical to less reactive forms prevent its reaction with GA the entire quantity of which is used for radicals scavenging. This result shows the role of time parameter in systems where substrates are engaged in various reactions taking place simultaneously. The inactivation of 1,1-diphenyl-2-picrylhydrazyl radical by studied antioxidants in homogeneous system confirmed observations made on the basis of lipid layer properties indicating their antagonistic action (at least at studied conditions). Formation of layers in post-oxidation situation did not depend whether tocopherol was oxidized during oxidation of lipid/tocopherol mixture or was introduced as pre-oxidized. This may be interpreted as indication that products of tocopherol oxidation may stabilize lipid layers.

## Introduction

Environmental stress (drought, salinity, low/high temperature, excess of light) causes an increase in the concentrations of reactive oxygen species (ROS) in the cell. Short-term or chronic presence of ROS excess is called oxidative stress, and its existence can lead to serious damage or death of cells. Under stress conditions, lipids building the cell membranes constitute the first target for ROS whose action leads to the disturbances or loss of membrane functions. Peroxidation-induced changes in lipid composition result in the alteration of mechanical properties of membranes which in turn can disrupt the arrangement of the individual components (disorder of the membrane proteins), influence ion transport (gated ion channels), and modify selectivity and permeability of membranes (Frankel 2005).

Defense reaction to stress is the synthesis of antioxidants whose quantity significantly increases and their uneven distribution can lead to locally high concentrations. Multiple literature data confirm rise in an amount of both hydrophilic (Greń et al. 2012; Łabanowska et al. 2012; Grzesiak et al. 2013; Marcińska et al. 2013; Barbasz et al. 2015) and hydrophobic (Havaux et al. 2000; Munné-Bosch and Alegre 2000; Gzyl-Malcher et al. 2010) antioxidants under stress conditions.

The antioxidant actions of polyphenols have been recognized a long time ago. These substances are recommended for both preventive and intervention use (Watson et al. 2013).

Tocopherols play a special role in the protection of plant lipid membranes against oxidation. Each of these substances works in its characteristic medium: tocopherols—in a hydrophobic hydrocarbon inner part of the membrane; gallic acid (GA)—a representative of polyphenols—soluble and reactive in aqueous media. Potential synergy of both compounds should, therefore, be particularly characteristic for cell membranes constituting the contact zone of the two environments.

There are different interpretations of the effects observed for systems where two various antioxidants operate simultaneously. In many papers, it was proved that polyphenolic co-antioxidants can reduce tocopheroxyl radical formed in the reaction with oxidizing radicals. Results of kinetic studies of oxidation of linoleic acid incorporated into SDS micelles in the presence of TOH and polyphenols (where peroxidation was initiated by water-soluble azo initiator 2,2′-azobis(2-amidinopropane) hydrochloride (AAPH)) indicated the possibility of regeneration of TOH by studied green tea polyphenols. The largest effect was found for GA suggesting synergistic action of these antioxidants (Zhou et al. 2005; Dai et al. 2008). Regeneration of TOH by phenolic compounds was also found in homogeneous system (Pazos et al. 2007).

TOH acting separately may cause opposite effects depending on conditions and system structure and composition. In addition to the widely acclaimed antioxidant action, there are indications of the pro-oxidative action of tocopherol radical formed in oxidizing conditions (Bowry and Stocker 1993; Min and Kim 2008). By considering oxidative potentials of redox systems, it was noticed that tocopherol radical can act pro-oxidatively when it remains in the system (Buettner 2006). In view of what stated above, it was even suggested that TOH can exhibit antioxidant properties only in presence of co-antioxidant (Bowry and Stocker 1993; Zhou et al. 2000).

Possible mutual action of various antioxidants was discussed in terms of thermodynamic (O–H bond dissociation enthalpy) and kinetic parameters of reactions taking place in such multicomponent systems (Pazos et al. 2007; Hoelz et al. 2010; Amorati et al. 2002, 2003). Antioxidative action can be realized by transfer of either hydrogen atom or free electron between reactants (Dorović et al. 2014; Ji et al. 2006). Thus, when comparing the antioxidant ability ionization potentials and enthalpy of O–H bond dissociation (BDE) are considered as the very important quantities (Leopoldini et al. 2011). It was proved (Fujisawa et al. 2006) that co-antioxidant can effectively regenerate tocopherol when its BDE is lower or similar to BDE of tocopherol molecule. In studies of interactions of various flavonoids with tocopherol, these authors showed that depending on flavonoid structure either synergistic effect or lowering of antioxidant capabilities are possible. Also in other papers, authors correlate antioxidative action in systems containing two antioxidants with BDE and ionization potentials of co-antioxidant molecules determined by their chemical structure (Hoelz et al. 2010; Fujisawa et al. 2006; Mukai et al. 2005; Kadoma et al. 2006).

Quantitative and qualitative composition and microenvironment specificity of the reaction medium represent also factors strongly influencing the effectivity of cooperation of various antioxidants (Min and Kim 2008; Mukai et al. 2005).

The aim of the work was to study, in model systems, the effect of simultaneous presence of TOH and GA on galactolipids’ oxidation induced by ozone dissolved in aqueous medium. In natural systems, galactolipids are the most common lipids in nature constituting a major component of the membranes of chloroplasts providing the environment for membrane proteins involved in photosynthesis. ROS can be formed during photosynthesis, and thus, chloroplasts are their main generator. The studies are a continuation of the previous works (Rudolphi-Skórska et al. 2014; Gzyl-Malcher et al. 2008a, b) and especially of Rudolphi-Skórska et al. (2014) where modification of the physical properties of the galactolipid layers caused by oxidation taking place in situ and protection action of TOH were examined.

## Materials and Methods

### Materials

Galactolipids: mono- (MGDG) and digalactosyldiacylglycerol (DGDG) were from Avanti Polar Lipids Inc. (USA/Canada).

α-Tocopherol (TOH) of ≥96 % purity and 1,1-Diphenyl-2-picrylhydrazyl radical (DPPH) were obtained from Sigma.

Phosphate salts used for buffer preparation were of chemical purity, POCH (Poland). Solvents (chloroform, ethanol) of chemical purity were from POCH (Poland).

Freshly deionized water was produced by HLP 5 apparatus Hydrolab (Poland).

10 mM phosphate buffer pH 7 was used in all experiments.

### Methods

#### Methods of Ozonation and Determination of Ozone Concentration

Ozone generator FM 500 (Grekos, Poland) supplied by oxygen, working on the principle of corona discharge was used as a source of ozone. The yield of ozone production was in the range 200–500 mg h^−1^. Gas mixture coming from the ozone generator was bubbled through a scrubber containing sodium carbonate solution of high pH to remove traces of nitrogen oxides which can be formed during the electric discharges. Buffer solution was saturated with ozone containing gas. Defined ozone concentrations were obtained by the appropriate dilution of the stock ozone solution.

Ozone concentration in buffer was determined by classical indygo method based on the decrease in absorbance of indigo carmine (sodium indigodisulfonate) at *λ* = 287 nm (Bader and Hoigne 1981, 1982; Majewski 2012). Discoloration of the dye was calibrated versus direct ozone UV peak at 250 nm used as a primary standard with molar extinction coefficient equal to 3200 M^−1^ cm^−1^ (Sonntag and Gunten 2012). As ozone content in aqueous solutions decreases very fast due to multiple reactions, samples of solutions for analysis were taken at the moment of lipid deposition.

#### Surface Pressure Isotherms

Surface pressure isotherms were obtained using Langmuir trough (Minitrough, KSV, Finland) of total surface area 243 cm^2^ with Pt-Wilhelmy plate used for surface tension detection. Measurements were performed at temperature of 25 °C at a constant rate of barrier movement equal to 5 mm min^−1^ which, for the most of the experiments, corresponded to the rate of area per molecule (***A***) decrease in the range 2–4 Å^2^ molecule^−1^ min^−1^.

#### Anti-DPPH Radical Activity Determination

The radical scavenging activities of studied antioxidants were determined for the reaction with a free radical probe—DPPH. Ethanolic solutions of antioxidants of concentrations in the range 6.6 × 10^−6^–1.3 × 10^−5^ and three ratios of TOH to GA in mixtures were used in measurements. The decrease in absorbance of DPPH band at 517 nm was measured for 5 min at room temperature. The anti-DPPH radical activity of antioxidants was calculated as the percentage reduction of radical concentration, *Q* (quenching) defined as

$$ Q = 100(A_{0} - A_{\text{a}} )/A_{0}, $$

where *A*_0_ is the initial absorbance of DPPH solution and *A*_a_ is the value for the mixture of DPPH and antioxidant (Molyneux 2004).

## Results

### α-Tocopherol

**Fig. 1:**
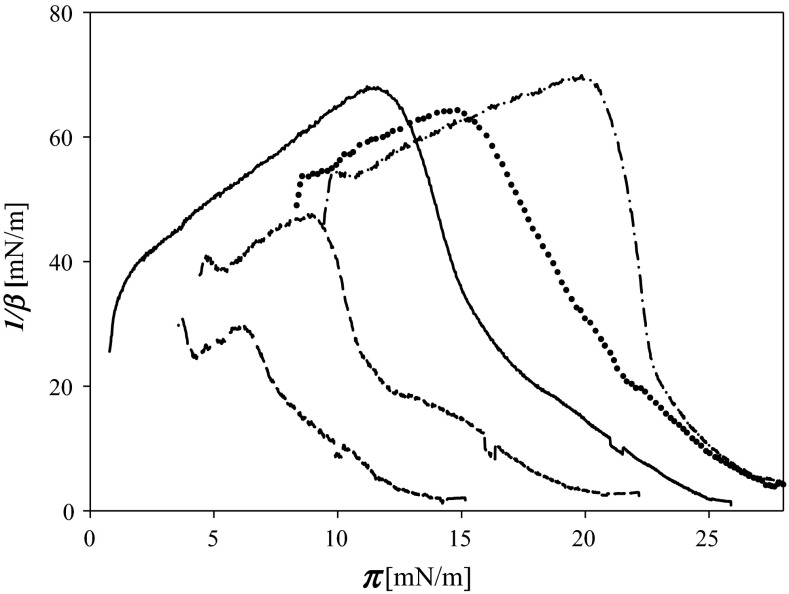
Compressibility modulus 1/*β* of α-tocopherol layers oxidized to the various degrees as a function of surface pressure ***π***. Οzone concentrations in the subphase: *solid line* 0; *short dash* 0.08 ppm; *long dash* 0.095 ppm; *dotted* 0.11 ppm; *dash–dot* 0.26 ppm

A significant number of the experiments performed with TOH spread on aqueous subphase (10^−2^ M PBS pH 7) confirmed well-known ability of the compound to undergo oxidation, e.g., by oxygen from the air. Even newly purchased samples showed some degree of oxidation. Therefore, it was necessary to find a parameter that would be a sensitive probe of quality of the compound used in experiments. As described in previous work (Rudolphi-Skórska et al. 2014), the surface pressure increase associated with the appearance of charged oxidation products is too small (when at low oxidation degree) to be used for this purpose. After analyzing surface pressure isotherms obtained for various TOH samples under various conditions, the dependence of static compressibility modulus (1/*β*) on surface pressure can be recommended as a probe of the sample quality. Non-monotonic behavior of this dependence (Fig. 1) ensures high sensitivity of the evaluation of the degree of sample oxidation.

At small oxidation degree TOH layer becomes very compressible and passes through collapse at very low surface pressures. Only at higher extent of oxidation, the layer becomes more resistant to compression and inflection point of the surface pressure isotherm (corresponding to the maximum of 1/*β* values) shifts to higher values of surface pressure. The layer formed from the “best” (oxidized to smallest degree, if any) is characterized by maximum of the coordinates: 1/*β* ≈ 70 mN m^−1^ reached at *π* ≈ 11 mN m^−1^.

To determine conditions under which oxidation occurs most efficiently and accepting the compressibility factor as a sensor of an extent of this reaction many experiments were performed in which oxidation was done by

- ozone at defined concentration in aqueous phase but acting on layers of various surface densities of TOH molecules,
- ozone administered as gas directly to the housing of Langmuir trough,^1^
- ozone acting from a gas phase on dry TOH film deposited on the walls of glass cell.

By analyzing 1/*β* = *f*(***π***) dependence, it was found that oxidation degree depended on the accessibility of TOH molecules for ozone. When TOH layer was oxidized by ozone dissolved at defined concentration in aqueous subphase, the degree of oxidation was greater when the initial two-dimensional density of TOH layer was smaller (data not shown). Oxidation of a dry TOH film by application of ozone from a gas phase occurred to be quite unrepeatable as the result of non-reproducibility of the formation of TOH film on glass walls by drying (which gives layers of accidental thickness). Due to that also mutual accessibility of substrates was also uncontrollable. The highest oxidation degree was reached when diluted layer of TOH spread on aqueous subphase was oxidized by ozone supplied from the gas phase.

Before studying the effect of GA on oxidation of galactolipids and tocopherol, it was proved that at applied GA concentrations, (below 10^−4^ M) its presence in aqueous subphase did not influence surface pressure isotherms neither that for TOH nor for MGDG and DGDG and galactolipid/tocopherol mixtures.

Oxidation of TOH and all other studied systems (galactolipids and their mixtures with TOH) was studied as a function of ozone concentration in the subphase (10^−2^ M PBS pH 7.0) in absence and when containing GA at two concentrations: 2 × 10^−6^ and 5 × 10^−6^ M.

**Fig. 2:**
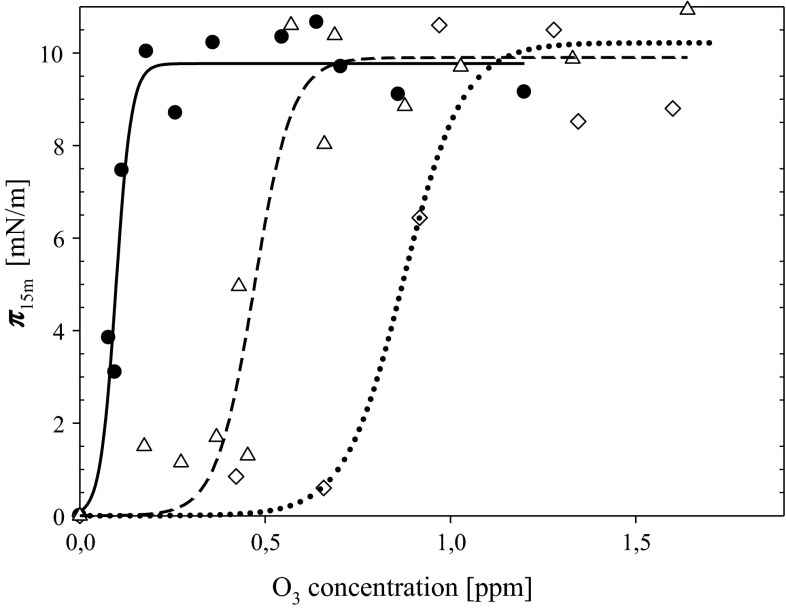
Surface pressure of α-tocopherol layer of density equal to 58 A^2^ molecule^−1^ reached after 15 min contact with subphase containing defined ozone concentration. *Solid line and filled circles* GA concentration in the subphase = 0, *dash line and triangle symbols* 2 × 10^−6^ M GA; *dotted line and diamond symbols* 5 × 10^−6^ M GA

The effect of TOH oxidation was presented by the dependence of ***π***_15m_—surface pressure values reached after 15 min contact of TOH layer of density 58 A^2^ molecule^−1^ (this is the lowest surface density of TOH exhibiting zero surface pressure in absence of ozone) spread on the subphase containing various ozone concentrations (Fig. 2). As was proved in previous paper with increased degree of TOH oxidation, the surface pressure of the layer increases due to appearance of charged oxidation products.

One can see (Fig. 2) that ***π***_15m_ parameter increases to a constant plateau level of about 10 mN m^−1^ regardless on the studied GA concentration. The presence of GA in the subphase causes a shift of this dependence proportional to GA level.

### MGDG and DGDG

**Fig. 3:**
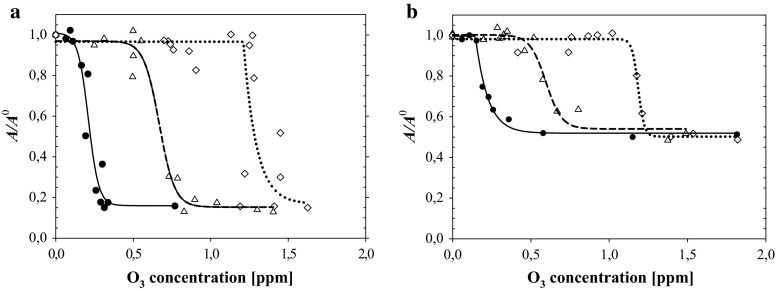
Ozone concentration dependence of the ratio ***A***/***A*** ^0^ lift-off values of galactolipid layers contacted ozone containing subphase (***A***) to the value characteristic for the layer contacting ozone-free subphase (***A*** ^0^). **a** MGDG, **b** DGDG. *Filled circles* GA = 0; *triangles* 2 × 10^−6^ M GA; *diamonds* 5 × 10^−6^ M GA

The effect of oxidation of galactolipids by dissolved ozone was presented (similarly as in Rudolphi-Skórska et al. 2014) by the ratio of ***A*** lift-off values (Abousalham et al. 2000) determined from surface pressure isotherms for oxidized lipid layers to that characteristic for non-oxidized galactolipid—***A***^0^ (Fig. 3).

Like in case of TOH oxidation, the presence of GA in the subphase causes the shift of ***A***/***A***^0^ = *f*(O_3_) dependencies towards higher ozone concentrations. It is worth to notice that for both studied galactolipids, the final state of oxidized layers reached at higher ozone concentrations did not depend on the presence and level of GA giving 85 and about 50 % decrease of the ratio of ***A*** lift-off values for MGDG and DGDG, respectively.

### Mixtures of MGDG and DGDG with TOH (1.7:1 M/M)

**Fig. 4:**
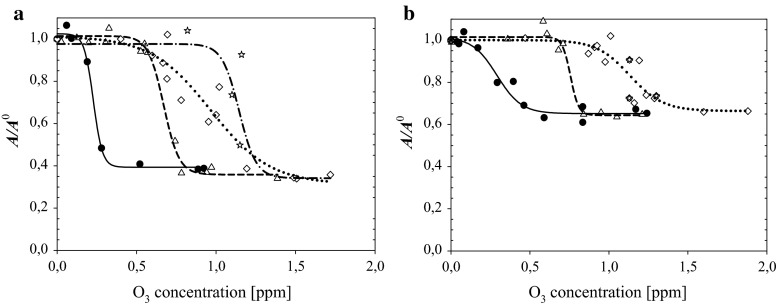
Ozone concentration dependence of the ratio ***A***/***A*** ^0^ lift-off values of mixed galactolipid/tocopherol layers contacted ozone containing subphase—(***A***) to the value corresponding the layer spread on ozone-free subphase (***A*** ^0^). **a** MGDG, **b** DGDG. *Filled circles and solid lines* GA = 0; *triangles and dash lines* 2 × 10^−6^ M GA; *diamonds and dotted lines* 5 × 10^−6^ M GA. Points marked as *stars* and *dash–dot corresponding line* represent results obtained for mixtures of galactolipids with pre-oxidized tocopherol

As described in (Rudolphi-Skórska et al. 2014) in the case of mixed galactolipid/TOH layers (at molar ratio 1.7:1), the amount of species left after oxidation at higher ozone concentrations (being capable of forming a monolayer) increased from 15 to about 40 % for MGDG and from about 50 to 65 % for DGDG. This effect occurred to be similar when GA was present in a subphase with the plateau levels of ***A***/***A***^0^ ratio being approximately the same independently on the studied GA additions (Fig. 4).

Analogously to one-component galactolipid layers, the presence of GA in subphase results in shifting the ***A***/***A***^0^ = *f*(O_3_) dependencies obtained for galactolipid/TOH mixtures. These changes can be described by determining ozone concentrations corresponding to the half height of curves fitted to the experimental points. The differences in these values are collected in Table 1 for all studied systems. They can be referenced to the theoretical maximal decrease of ozone level in aqueous solutions due to the bulk reaction with GA, assuming complete mineralization of GA: ${\text{C}}_{7}{\text{H}}_{6}{\text{O}}_{5} + 4{\text{O}}_{3} \rightarrow 7{\text{CO}}_{2} + 3{\text{H}}_{2}{\text{O}}$ (Carbajo et al. 2006).

**Table 1:** The differences in ozone concentrations shift ($\Delta c_{{{\text{O}}_{3} }}$) corresponding to the half height of curves obtained for two levels of GA in subphase divided by maximal possible ozone level decrease (assuming complete GA mineralization)

Layer composition	2 × 10^−6^ M GA		5 × 10^−6^ M GA	
	$\Delta c_{{{\text{O}}_{3} }}$ (ppm)	$\Delta c_{{{\text{O}}_{3} }}$ /0.384	$\Delta c_{{{\text{O}}_{3} }}$ (ppm)	$\Delta c_{{{\text{O}}_{3} }}$ /0.96
ΤΟΗ	0.35	0.91	0.77	*0.80*
MGDG	0.45	1.17	1.05	1.09
MGDG + TOH	0.44	1.14	0.67	*0.70*
MGDG + ΤΟΗ ox			0.90	0.94
DGDG	0.40	1.04	0.97	1.01
DGDG + ΤΟΗ	0.45	1.17	0.85	*0.88*

Italicized values indicate conditions where GA action is not limited to reduce ozone concentration by bulk reaction

At such stoichiometry: 1 mol GA reacts with 4 mol O_3_ what for the experimental conditions give the expected maximal displacements of ozone concentrations assuming that GA is not involved in any other processes:

$$ {\text{for}}\;2 \times 10^{ - 6} \,{\text{M}}\,{\text{GA}}\quad 0.384\,{\text{ppm}}\;{\text{O}}_{3}, $$

$$ {\text{for}}\;5 \times 10^{ - 6} \,{\text{M}}\,{\text{GA}}\quad 0.96\,{\text{ppm}}\;{\text{O}}_{3}. $$

The shift of ***π***_15m_ = *f*(O_3_) dependencies (adopted as an indicator of oxidation of tocopherol layer) due to the presence of 5 × 10^−6^ M GA is 20 % smaller than expected assuming stoichiometric intake of GA in bulk reaction with dissolved ozone. The presence of tocopherol in mixed layers with galactolipids results in a reduction in the shift of inflection points of ***A***/***A***^0^ = *f*(O_3_) relationships by approx. 30 and 12 % for MGDG and DGDG, respectively, at higher GA level (Table 1).

Peculiar effects, especially distinct for MGDG, were observed in the intermediate ozone concentrations at higher GA content (5 × 10^−6^ M) (Fig. 4). Exposition of lipid/TOH mixed layer to ozone changes the dependence of ***A***/***A***^0^, making it less steep in comparison to that obtained in absence and at lower GA level. However, when MGDG was mixed at the same ratio with pre-oxidized TOH sample (points marked as stars), almost parallel shift of ***A***/***A***^0^ = *f*(O_3_) function was obtained.

**Fig. 5:**
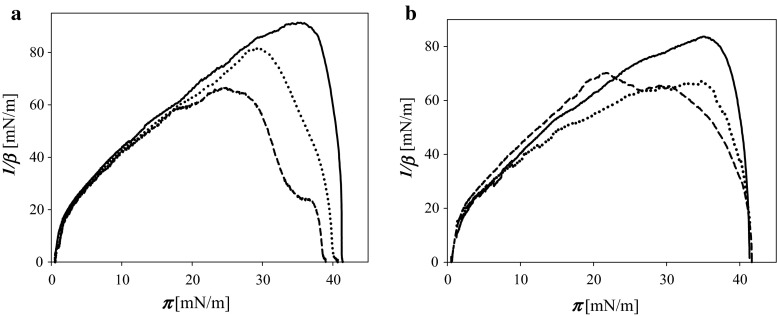
Compressibility modulus 1/*β* as a function of surface pressure ***π*** of layers: galactolipid—*solid line*, mixed galactolipid/TOH—*short dash*, galactolipid/pre-oxidized tocopherol—*dotted*, spread onto subphase not containing any ozone (PBS, pH 7.0). **a** MGDG, **b** DGDG

It was interesting to compare the effect of non- and pre-oxidized tocopherol on mechanical properties of mixed galactolipid/TOH layers expressed by compressibility modulus 1/*β* (Fig. 5).

The layer formed from the MGDG + TOH mixture at studied composition was less mechanically stable than one-component MGDG layer (breaking down at lower surface pressure). When oxidized tocopherol was mixed with the lipid, such layer was more resistant to compression (when comparing to the mixture of MGDG with non-oxidized tocopherol). Much smaller difference of compressibility lines was found for layers composed of DGDG and non- and pre-oxidized tocopherol.

For additional verification of anti- and co-antioxidant interaction determined on the basis of indirect effects in complex and unstable (fast ozone decomposition) system, the radical scavenging ability of TOH and GA and their mixtures against relatively stable DPPH radical probe was determined in homogeneous ethanol solutions. By monitoring a decay of DPPH radical concentration (measured as a decrease in absorbance of DPPH band), it was found that the effect obtained for GA + TOH mixtures was smaller than results calculated assuming simple additivity of the inhibition factor *Q* (Table 2).

**Table 2:** The inactivation of DPPH radicals by GA + TOH mixtures expressed by *Q* factor as compared to the sum of *Q* values determined for individual components

	*Q* factor exp. (%)	The sum of *Q* values of individual components	The relative deviation from additivity
GA/TOH molar ratio			
1:1	48.64	62.93	0.227
1:1.4	74.40	91.82	0.190
1:2	55.54	74.82	0.258
GA + TOH ox			
1:1	48.50	52.54	0.077
1:2	49.23	52.05	0.054

The relative deviation from additivity very little (if at all) depends on the GA/TOH molar ratio amounting on average 0.225.

## Discussion

Degradation of galactolipid layers due to oxidation expressed by the decrease of ***A***/***A***^0^ lift-off ratio can be treated as a sensitive sensor of ozone originated radical amount and activity. The effect of treatment of lipid layers with ozone and action of antioxidants can be divided onto two regions of applied oxidative stress (ozone concentration):

1. ozone concentration range above some threshold level where oxidation reaction produces the strongest modifications of layer composition and structure reflected by the greatest gradients of parameters;
2. the range of higher ozone content where the system stops responding to ozone level increase and virtually no further changes of layer parameters are detected.

The values of ozone concentrations corresponding to the half height of the dependencies of layer properties (taken as indicators of layer modifications) show that, as one can expect, TOH, as an antioxidant, is most sensitive to ozone presence (producing changes visible already at ozone concentration equal to 0.1 ppm). MGDG and DGDG exhibited higher but similar resistance to ozone with O_3 *h*/2_ ≅ 0.2 ppm. As shown in Fig. 3, GA presence causes the displacement of O_3 *h*/2_ values (Table 1) by an amount corresponding approximately to the stoichiometry of the reaction of GA mineralization. It means that in oxidation of galactolipid layers, the role of GA as a strong radical scavenger is limited to the diminishing of an amount of oxidizing radicals.

Results obtained for TOH (in form of layers or in homogeneous ethanol solutions) and galactolipid/TOH mixtures indicate that this simple effect of GA is modified in TOH presence. Already GA action in oxidation of tocopherol layers is different than its effect on the lipid oxidation. The shift of inflection points of ***π***_15m_ = *f*(O_3_) dependence is smaller (approximately by 20 % at higher GA concentration) than observed in the case of galactolipid oxidation and foreseen by the stoichiometry of GA + O_3_ reaction.

Similar diminishing of ozone concentration range (in comparison to the values expected when assuming the stoichiometric amount of ozone worn out in bulk reaction with GA) where galactolipids are effectively oxidized was found for mixed tocopherol/galactolipid layers at higher GA content (Fig. 4; Table 1). This decrease was especially distinct for MGDG/tocopherol mixture reaching about 30 %. The effectiveness of antiradical action of TOH/GA mixture in homogeneous system (inactivation of DPPH radical probe) occurred also to be smaller than the sum of effects of both components operating separately.

In explaining these observations, the interactions between two antioxidants have to be taken into account. As it was proved in many papers, GA is able to regenerate TOH from tocopheroxyl radical (Peyrat-Maillard et al. 2003; Karvela et al. 2012). Qualitative studies of Pazos (Pazos et al. 2007) showed that reduction of tocopheroxyl radical proceeds with a large excess of GA (of the order of hundreds). Thus, it seems reasonable to assume that some part of GA may be consumed in this reaction, however, regenerating only small amount of tocopherol. Similar approach was used by Peyrat-Maillard et al. (2003) in interpreting antagonistic effect of TOH and caffeic acid found for the reaction with radicals produced by spontaneous decomposition of AAPH. These authors correlated the negative deviation from additivity observed for the mixture of these antioxidants with estimated fraction of caffeic acid involved in the reduction of tocopheroxyl radical.

The effect of GA in oxidation of mixtures of MGDG with pre-oxidized TOH and in homogeneous inactivation of DPPH radicals showed that practically no deviation from additivity was registered. Mixed MGDG/TOHox layers formed on ozone containing subphase behaved in GA presence similarly as one-component galactolipid layer in the sense that ozone concentration range was displaced by the quantity corresponding almost exactly the stoichiometry of GA mineralization reaction. These observations indicate that GA is not involved in reduction of pre-oxidized TOH oppositely to situation when tocopherol mixed with galactolipid was oxidized in GA presence. One can conclude that GA can regenerate tocopheroxyl radical only when it is formed in situ (when both reactions proceed simultaneously). This hypothesis can be supported by literature data showing that intensity of EPR signal from tocopheroxyl radical decreases noticeably in time (Pazos et al. 2007) due to subsequent reactions. The authors underline the role of time between the moment of tocopheroxyl radical formation and GA addition. As it is known (Scott 1997), phenolic-type compounds by donating hydrogen from hydroxyl (–OH) group to free radicals become themselves relatively unreactive. The above information supports the interpretation of differences between the antioxidative roles of GA in the presence of non- and pre-oxidized tocopherol.

In the range of excess of oxidant, lack of further changes of the layer properties allows to assume that all system components were already transformed to its oxidized products. Results obtained in the absence and presence of GA show that the final state of oxidized galactolipid layers is insensitive to GA addition. As it was proved in Rudolphi-Skórska et al. (2014) and confirmed now in GA presence, MGDG undergoes much stronger deterioration due to oxidation in comparison to DGDG. TOH added to lipids at studied ratio caused an increase of the amount of post-reaction products capable for layer formation as expressed by the growth in ***A***/***A***^0^ ratio the value of which was the same despite GA presence and level (Fig. 4). Interestingly, galactolipids mixed with non- and pre-oxidized tocopherol exposed to higher ozone concentrations give products forming layers of similar properties (at least those determining the ***A***/***A***^0^ ratio). If growth of this value associated with tocopherol presence can be interpreted as an indication of increased post-oxidation layer stability, then one can say that this effect was the same regardless of whether tocopherol oxidation products were formed during the reaction with ozone or prepared separately and mixed with a lipid.

Comparison of the compressibility factors of mixed layers of galactolipids with non- and pre-oxidized tocopherol (Fig. 5) indicates that at least for MGDG, the mixed layer of lipid and pre-oxidized tocopherol was more resistant to compression (higher maximal 1/*β* value reached at higher surface pressure) than the layer containing non-oxidized tocopherol. This means that products of tocopherol oxidation appearing in the course of its antioxidant defense action can additionally stabilize lipid membranes.

## Conclusions

1. Oxidation of galactolipids by ozone dissolved in aqueous phase leads to degradation of lipid layers the degree of which (represented by the ratio of areas per molecule lift-off values of non-oxidized to oxidized layers) depends as sigmoidal-shape function on ozone concentration. GA shifts this dependence to higher ozone levels by the quantity corresponding to the stoichiometry of its reaction with ozone. Thus, the role of GA in galactolipid oxidation is limited to reducing the amount of oxidizing radicals. The character of lipid layers oxidized under conditions of oxidant excess did not depend on the absence/presence and the level of GA.
2. Layers of TOH in contact with GA solutions (at least at higher GA concentration) respond to ozone presence at lower concentrations than expected assuming the complete intake of GA in bulk reaction with ozone. This indicates that noticeable amount of GA may be involved in the reaction of tocopheroxyl radical reduction.
3. In the case of galactolipid/tocopherol mixed layers (of 1.7:1 molar ratio), GA protective action (at its higher level) was smaller than found for one-component lipid and than that expected taking into account stoichiometry of GA mineralization. This was interpreted in terms of involvement of GA in the reaction of tocopherol regeneration. The final post-oxidative state of layers (in the range of higher ozone concentrations) was insensitive neither to GA presence and level nor to that whether tocopherol underwent oxidation being a component of the layer exposed to ozone present in subphase or separately pre-oxidized and mixed with lipid.
4. Different reactions of layers of galactolipids mixed with non- and pre-oxidized tocopherol to ozone of intermediate concentrations (corresponding to the biggest changes of layers’ properties) especially distinct for MGDG at higher GA level, indicate that the involvement of GA in reaction with tocopheroxyl radical depended on its life time. GA was consumed in the reaction with tocopheroxyl radical only when the latter was formed in situ. This conclusion may be of some importance when estimating antioxidants’ protective ability by paying attention to time parameter.
5. Results of compressibility modulus shown that at least MGDG/pre-oxidized TOH mixed layers were more resistant to compression than layers containing non-oxidized tocopherol. Thus, one can conclude that oxidized tocopherol can modify lipid layers’ properties influencing their stability and stiffness.
6. The impact and interactions of studied antioxidants in protection of lipid layers situated at the aqueous solution/air interface were compared with their ability to inactivate the radical probe in homogeneous system. Negative deviation from additivity found for GA and TOH mixtures reacting with DPPH radicals confirms the conclusions derived from studies of monolayers’ oxidation.

## Footnotes

1 In the previous paper (Rudolphi-Skórska et al. 2014), the concentration of ozone in gaseous phase was erroneously given as 1–3 ppm, whereas it was 100-fold higher being in the range of 100–300 ppm.
